# Parallel Changes in Harvey-Bradshaw Index, TNF*α*, and Intestinal Fatty Acid Binding Protein in Response to Infliximab in Crohn's Disease

**DOI:** 10.1155/2017/1745918

**Published:** 2017-10-23

**Authors:** Anas Kh. Al-Saffar, Carl Hampus Meijer, Venkata Ram Gannavarapu, Gustav Hall, Yichen Li, Hetzel O. Diaz Tartera, Mikael Lördal, Tryggve Ljung, Per M. Hellström, Dominic-Luc Webb

**Affiliations:** ^1^Department of Medical Sciences, Gastroenterology and Hepatology Unit, Uppsala University, Uppsala, Sweden; ^2^Department of Medicine, Division of Gastroenterology and Hepatology, Danderyds Sjukhus, Danderyd, Sweden; ^3^Abbvie, Solna, Sweden

## Abstract

Intestinal fatty acid binding protein (I-FABP) indicates barrier integrity. Aims: determine if I-FABP is elevated in active Crohn's disease (CD) and if I-FABP parallels anti-TNF*α* antibody (infliximab) induced lowering of TNF*α* and Harvey-Bradshaw Index (HBI) as potential indicator of mucosal healing. I-FABP distribution along human gut was determined. Serum from 10 CD patients collected during first three consecutive infliximab treatments with matched pretreatment and follow-up samples one week after each treatment and corresponding HBI data were analyzed. I-FABP reference interval was established from 31 healthy subjects with normal gut permeability. I-FABP and TNF*α* were measured by ELISA; CRP was measured by nephelometry. Healthy tissue was used for I-FABP immunohistochemistry. Pretreatment CD patient TNF*α* was 1.6-fold higher than in-house reference interval, while I-FABP was 2.5-fold higher, which lowered at follow-ups. Combining all 30 infusion/follow-up pairs also revealed changes in I-FABP. HBI followed this pattern; CRP declined gradually. I-FABP was expressed in epithelium of stomach, jejunum, ileum, and colon, with the highest expression in jejunum and ileum. I-FABP is elevated in active CD with a magnitude comparable to TNF*α*. Parallel infliximab effects on TNF*α*, HBI, and I-FABP were found. I-FABP may be useful as an intestine selective prognostic marker in CD.

## 1. Introduction

The gastrointestinal (GI) tract maintains a barrier against harmful agents (e.g., xenobiotics) by means of the epithelial mucosa, which is compromised in many disorders and/or diseases. This defense barrier is comprised of a single layer of columnar epithelial cells lining the lumen. It possesses a regulated permeability function by way of tight junctions as well as a mucopolysaccharide covering. Through these, ingested nutrients should pass [1, 2]. Factors (e.g., environmental and dietary, particulate matter, high-fat diet) that damage enterocytes compromise intestinal immune defenses [3, 4]. This damage must be continuously repaired. Impaired barrier function is suspected of increasing the risk for progression to debilitating inflammatory conditions, such as inflammatory bowel diseases (IBD), including Crohn's disease (CD) [1, 5]. According to Montreal phenotype classification, unlike ulcerative colitis (UC), the inflammatory site in CD can occur at any part of the GI tract, characterized by segmental transmural inflammation, with more pronounced necrosis of the affected epithelium having the potential of manifesting extraintestinal immunopathy-associated symptoms [6].

Current CD monitoring focuses on fecal (e.g., calprotectin) or circulating inflammatory (e.g., C-reactive protein (CRP)) markers, imaging and self-reporting assessment, such as the Harvey-Bradshaw Index (HBI) [7, 8]. Current validated diagnostic practices include full ileocolonoscopy with biopsies, radiology with contrast media, and magnetic resonance imaging, which are costly and cumbersome to patients and are not generally performed for monitoring purposes. X-ray, for example, requires specialized facilities and is not recommended for CD monitoring due to high cumulative ionizing radiation [6]. There is a need for less cumbersome, noninvasive, and specific CD monitoring that has driven the search for new biomarkers to accurately and selectively reflect treatment outcomes and GI tract mucosal healing.

Human I-FABP is a 132 amino acid 15.2 kDa cytoplasmic protein that is highly expressed in intestinal epithelial cells. It is released into blood circulation upon intestinal injury, with serum levels increasing within 30 min of intestinal damage (e.g., mesenteric ischemia) [9]. Elevated urinary I-FABP level correlates positively with defective epithelial mucosal integrity, elevated intestinal permeability, and high CRP-associated gastrointestinal bacterial translocation [10]. Upon increased turnover of enterocytes, blood I-FABP increases. It also has a short half-life (approx. 11 min) in humans [11], thus indicating the immediate status of the GI mucosal barrier. It was reasoned that I-FABP might fluctuate in active CD at pace with relapse-remission cycles, potentially serving as a biomarker of mucosal healing to monitor treatment outcomes.

The main hypothesis of this study tested if I-FABP has potential to be used for acute monitoring of epithelial mucosal integrity in CD that indicates treatment outcome. The aims were to determine (1) if I-FABP is elevated in active CD and (2) if I-FABP parallels infliximab induced lowering of circulating TNF*α* and HBI as a more direct estimate of mucosal healing and to characterize I-FABP expression along with the human GI tract and relate this to mucosal area.

## 2. Materials and Methods

### 2.1. Human Subjects

A database containing a total of 47 CD patients with repeat visits that underwent infliximab therapy (Remicade®, 5 mg/kg body weight) between years 2000 and 2005 was searched for matched biobanked serum samples from naïve pretreatment before infliximab infusion on the first day of treatment and another two consecutive infliximab infusions as well as matching follow-up samples, each one week after every infusion along with corresponding HBI data (i.e., 6 visits for each subject for which serum and other data was available). Samples from 11 CD patients were identified that fulfilled these requirements. One was excluded due to presence of antidrug antibodies already at the first time point (unpublished results), resulting in *n* = 10 CD patients in this study. Colonoscopy was performed to document colonic inflammation in all patients. Two of these included ileoscopy, in which ileocecal involvement was further documented. Ileocecal involvement in the other 8 subjects was not investigated and therefore cannot be ruled out. To establish a reference interval for serum I-FABP, samples from 31 healthy adult controls with normal gut permeability assessed by lactulose : mannitol ratio ≤ 0.7 [12] were included. Another 61 healthy adult controls, constituting an established in-house TNF*α* reference interval was used for comparison against TNF*α* in the CD patient samples.

### 2.2. Blood Samples

**Figure 1:**
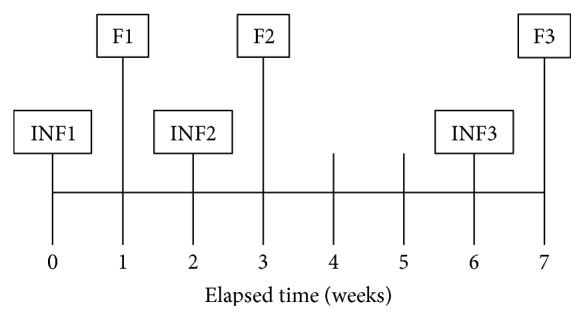
Time points for the 3 consecutive infliximab infusions (Inf) and weekly follow-ups (F). At each visit, serum for TNF*α*, I-FABP, and CRP was obtained along with HBI data.

Blood samples were taken on days 1, 14, and 42 immediately before infliximab infusion and on follow-up visits, each one week after infusion (Figure 1). On the first blood draw for serum, immediately before infusion 1 (Inf1), CD patients were naïve to infliximab. This time point was used as a baseline to normalize data. To verify a drug (i.e., infliximab) effect in relation to circulating I-FABP, TNF*α* and CRP levels were measured.

A 50X protease inhibitor cocktail solution was prepared by dissolving a SigmaFast tablet (catalog number S-8830, Sigma-Aldrich, USA) in 2.2 ml deionized H_2_O and adding 5.5 *μ*l 10 mM KR-62436 (catalog number K4264, Sigma-Aldrich) in DMSO along with a separate 68 mM 10X EDTA stock [13]. After vortexing, the 50X cocktail was pipetted immediately into blood tubes to 1X final concentration. Because this cocktail was not added at the time of blood draw in the case of the biobanked infliximab infusion and follow-up samples, it was added along with final 1X EDTA (for comparisons to plasma) prior to thawing in order to minimize degradation during or subsequent to thawing and to permit identical chemical composition as with all the recently obtained samples (i.e., controls) they were compared against, into which this cocktail was added at the time of blood draw.

### 2.3. Enzyme Linked Immunosorbent Assay (ELISA)

Serum I-FABP was measured by commercial research ELISA kit (catalog number HK406-2, Hycult Biotech, Uden, The Netherlands) according to the product insert using 20-fold sample dilution. TNF*α* and IL-6 were measured using “V-PLEX” ELISA kits (Meso Scale Discovery, Rockville, MD, USA). The V-PLEX kits are validated kits produced for high reproducibility between lots. IL-6 was included because it is thought to drive CRP production and release from the liver. Serum CRP, a routine clinical chemistry biomarker of inflammation, was measured using CRP Vario 6K26 assay (Sentinel CH. SpA Via Robert Koch, 2 Milan 20152 Italy) on an Architect analyzer (Abbott Labs, IL, USA) at the clinical chemistry department, Uppsala University Hospital.

### 2.4. Immunohistochemistry

Paraffin-embedded transmural sections (4 *μ*m thickness) of normal human stomach (cardia, corpus, and fundus), small intestine (jejunum and ileum), and colon were obtained from nonpathological surrounding tissue from donors undergoing different GI surgeries (colectomy or others, e.g., bariatric surgery). The duodenum could not be obtained for this study. Immunostaining was performed using alkaline phosphatase-FastRed detection of rabbit polyclonal primary antibodies against human I-FABP (catalog number HP9020, 1 : 50 dilution, Hycult Biotech). Western blotting has been shown to yield a single band with this antibody [14]. Two of the authors (AKhA and VRG) assessed staining independently, which was scored from 0 to ++++ for number of positive epithelial cells and for intensity of staining. (*n* = 3 slides for each GI segment from different subjects versus negative controls). Visual scoring was translated to relative concentrations by comparison against different concentrations of dye solutions quantified by absorbance. The concentration difference between one visual score and the next was found to be approximately fourfold. These results were then used to calculate I-FABP relative abundance along with the GI tract using the literature findings for GI tract surface area of the epithelium [15] and I-FABP expression in duodenum [16].

### 2.5. Statistics

Results are expressed as mean ± standard error of mean (SEM) unless otherwise stated. The threshold for significance was set to *P* < 0.05. Paired *t*-test was used to compare the difference in the levels of I-FABP and TNF*α* between the infusion and follow-up days. Mann–Whitney *U* test was used to compare the I-FABP levels in CD patients versus healthy controls. Statistical analysis was done using SigmaPlot software (ver. 11.0). Power analysis was done using “R” software (http://www.r-project.org) to calculate optimal sample size.

### 2.6. Ethical Consideration

The ethical approval number is Dnr: 92 : 38 for CD patients (Karolinska Institute, Sweden). Healthy subjects were further covered under Dnr: 2012/323 (blood samples), 2010/157, and 2010/184 (surgical specimens for immunohistochemistry) to Uppsala University.

## 3. Results

### 3.1. CD Patient Characterization

CD patient characteristics are presented in Table 1. CD patients number 3 and number 10 reported normal HBI at onset of the study (Inf1). However, their CRP (20 and 8.1 mg/L) and TNF*α* (2.32 and 3.03 ng/L) levels were in the upper 50% among CD patients and well above healthy controls.

### 3.2. I-FABP Is Elevated in Active CD and Parallels TNF*α* and HBI

**Figure 2:**
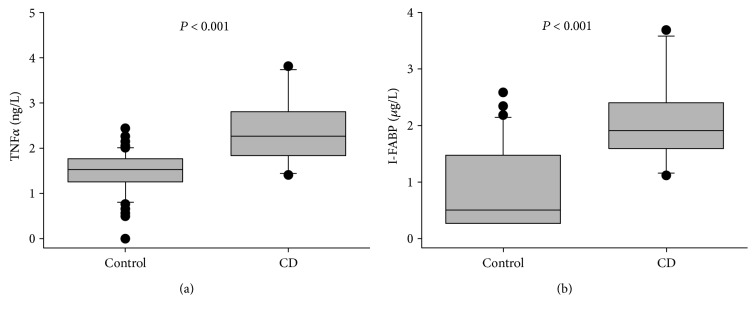
I-FABP is increased in CD to a higher magnitude than TNF*α*. (a) TNF*α* was higher (1.6-fold) in the CD patient group (*n* = 10) prior to infliximab therapy than the healthy adult control reference group (*n* = 61). (b) I-FABP was also higher (2.5-fold) in CD patients prior to infliximab therapy compared to the healthy adult control group (*n* = 31).

Pretreatment TNF*α* and I-FABP levels were examined (Figure 2). TNF*α* levels of CD patients averaged 1.6-fold higher than those of the 61 healthy controls (2.34 ± 0.22 versus 1.48 ± 0.06 ng/L, *P* < 0.001) when using the Mann–Whitney *U* test (Figure 2(a)). The TNF*α* reference interval was 0.51–2.26 ng/L (5–95% percentile). The I-FABP serum levels of CD patients were 2.5-fold (2.07 ± 0.23 versus 0.84 ± 0.13 *μ*g/L, *P* < 0.001) higher than those of the healthy controls which was established in-house as a local reference group (Figure 2(b)) with reference intervals 0.24, 1.45, and 2.43 *μ*g/L (5, 75 and 95% percentile). In 9 of the 10 CD patients, I-FABP was initially above this 75% percentile; 2 of these 9 were also above the 95% percentile. For comparison, 8 patients had TNF*α* above the 75% percentile; 5 were above the 95% percentile (see Table 1).

In control experiments on different occasions when plasma and serum were collected at the same time from the same subjects, it was found that plasma I-FABP values were consistently 2–4-fold lower than serum. In spike recovery experiments, the protease inhibitor cocktail used here yielded higher values in plasma and serum. Immediate addition of this cocktail at the time of blood draw, done for controls in this paper, protected I-FABP. Without it, serum I-FABP was 15% lower (*n* = 6, *P* < 0.05), suggesting losses can occur even during one hour that typically elapses from blood draw until freezing. This apparent protection against proteolytic degradation was only partial; I-FABP declined in plasma and serum to 70% and 50% after storage at room temperature for 24 h (*p* = 0.0001). Spiking experiments yielded similar results. No losses were identified in relation to repetitive freeze-thaw itself. The biobanked samples were frozen only once and received the cocktail prior to thawing. The I-FABP concentrations for the CD patients could have been 15% higher had this cocktail been used when the samples were first drawn (i.e., prior to biobanking).

**Figure 3:**
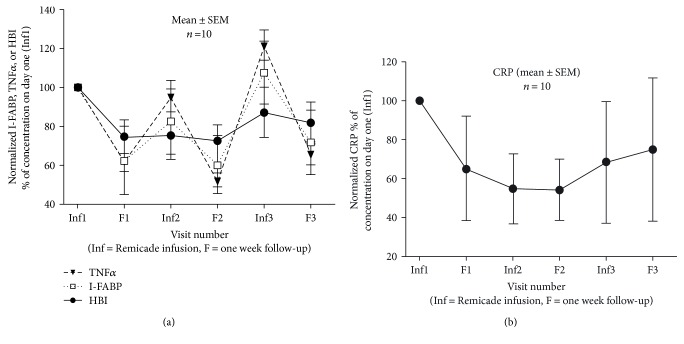
(a) I-FABP parallels TNF*α* and HBI during infliximab therapy. Data is normalized to values at first visit (Inf1) in serum collected immediately prior to the first infliximab infusion. Each data point is mean ± SEM, *n* = 10 CD patients; (b) CRP declines during infliximab therapy. Data is normalized to values at the first visit (Inf1) in serum collected immediately prior to the first infliximab infusion. Each data point is mean ± SEM, *n* = 10 CD patients.

TNF*α* at F1 was lower than Inf1 as well as subsequent infliximab infusion day Inf2, demonstrating that the drug effect was clearly identifiable with significance *P* = 0.001 using ANOVA on ranks (Figure 3()). This was transient, returning to levels of Inf1 by the time of Inf2. HBI followed the same trend, although the peaks and troughs were less pronounced. The mean I-FABP level was also lower at F1 than Inf1 (*P* = 0.019) while it did not reach significance at F2 and F3 relative to Inf2 and Inf3 (*P* = 0.420 and 0.229). No significance was found (*P* = 0.180) between Inf1 and F3 (Figure 3(a)). This could be due to the lack of adjustment for the infusion dose of Remicade to TNF*α* levels in blood. Power analysis indicated a sample size of 25 to be optimal to reach significance for the range of values obtained in the present dataset. Combining all data (all three infusion and follow-up pairs) to reach *n* = 30, statistical significance was reached using a paired *t*-test (*P* = 0.014).

CRP concentrations declined gradually during infliximab treatment (Figure 3(b)), with F1 versus Inf1 reaching significance (*P* = 0.037). A significant decrease was also found (*P* = 0.041) at F3 (2.3 ± 0.8 mg/L) relative to Inf1 (11.7 ± 5.6 mg/L). No significance was found between the combined infusion and follow-up pairs for all 3 visits (*P* = 0.510). When CRP data was sorted as increase, decrease, or no change (cut-off > or <10% from preceding infusion day value), a clear pattern emerged. In all 30 cases, TNF*α* declined on follow-up (i.e., no cases with increase or no change). In 18 cases (60%), I-FABP also declined, as did IL-6 and CRP. Only two additional cases had lower IL-6. In 12 cases (40%), neither I-FABP nor CRP declined, which was so for IL-6 in 10 of these cases.

### 3.3. I-FABP Is Expressed throughout the Human GI Tract, Including the Ileum and Colon

**Figure 4:**
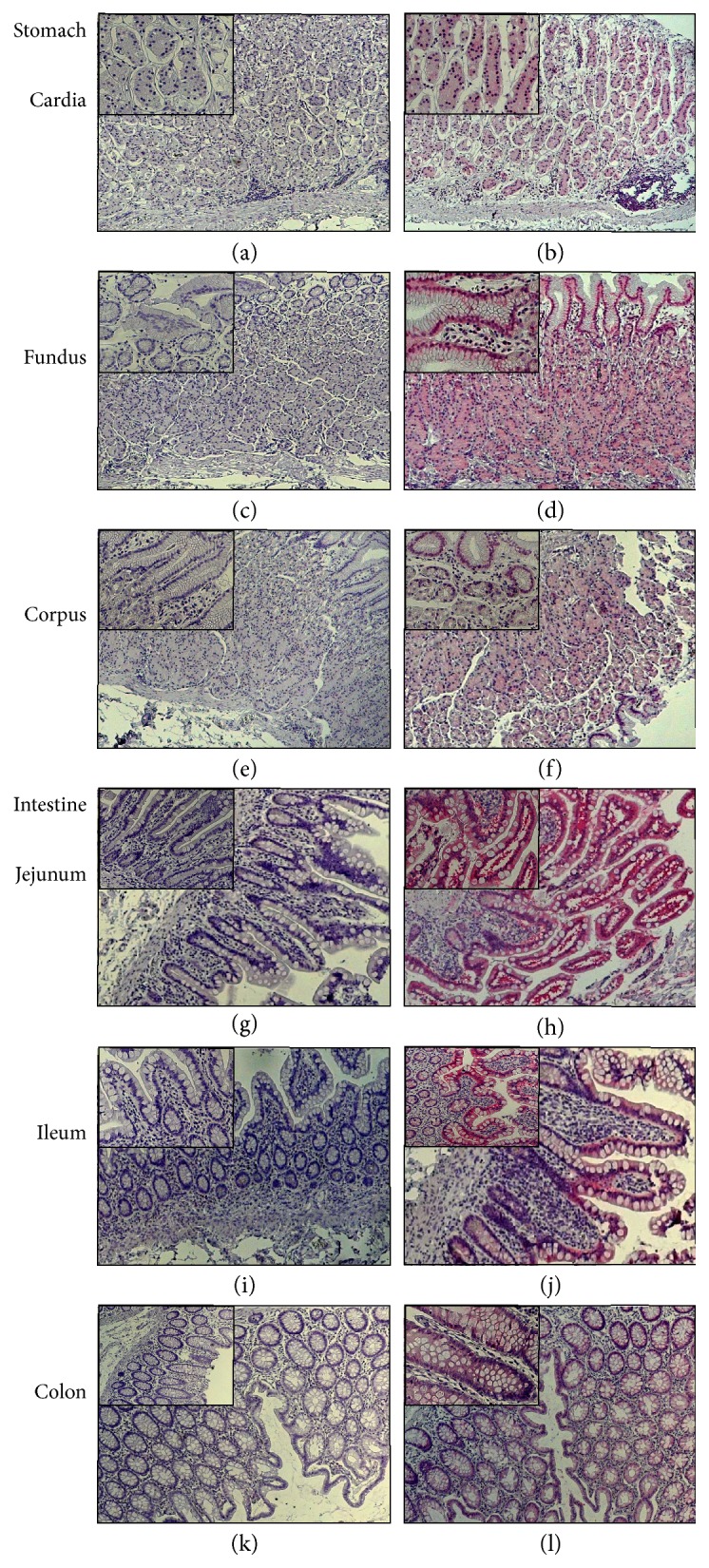
I-FABP immunoreactivity along human GI tract, including ileum and colon. Immunohistochemistry was performed on the cardia, fundus, corpus, jejunum, ileum, and colon. (a), (c), (e), (g), (i), and (k) are negative controls corresponding to I-FABP immunoreactivity using alkaline phosphatase-FastRed staining in (b), (d), (f), (h), (j), and (l). Images are representatives from 4 slides for each intestinal segment, 1 negative control, and 3 receiving I-FABP antibody. FastRed (pinkish red) staining indicates positive immunoreactivity. For the number of mucosal epithelium cells, a score of ++++ was assigned as a result of no negative epithelial cells being found. Results are itemized in Table 2. Images are with 5x objectives in order to reveal the entire transmural sections. Insets are 10x in order to highlight the fact that essentially all mucosal epithelial cells were immunoreactive for I-FABP.

Immunohistochemistry showed selective I-FABP immunoreactivity confined to the epithelium of the stomach (cardia, fundus, and corpus), small intestine (jejunum and ileum), and colon (Figure 4). The strongest immunoreactivity (i.e., highest protein levels) occurred equally in the jejunum and ileum, followed by the colon. The I-FABP immunoreactivity was confined to the mucosal epithelium, with no observable staining in other layers (lamina propria, smooth muscle, enteric neurons, blood vessel endothelium, etc.).

Estimations of relative abundances by GI segments are provided in Table 2. When relative sizes of each segment were taken into consideration, roughly 45–48% of I-FABP abundance can be expected equally for jejunum and ileum, with 1.6–4.5% occurring in the colon.

## 4. Discussion

Our main purpose was to determine if treatment outcomes could be monitored with I-FABP in the context of mucosal healing estimation in active CD patients. Elevated I-FABP levels represent shedding of cytosolic contents, reflecting the balance of enterocytic death, turnover, and healing. The I-FABP levels were initially elevated in CD patients when compared to healthy subjects and declined with infliximab therapy, which is consistent with indicating mucosal healing and treatment outcomes.

I-FABP paralleled TNF*α* and HBI, while CRP slowly declined during two months. I-FABP measurements could be of benefit for monitoring relapse and remission (i.e., mucosal healing) cycles, which is a cardinal feature of CD. These parallel I-FABP and TNF*α* patterns are consistent with the concept of I-FABP measurements supplementing TNF*α* measurements to gain more complete and intestinal-specific CD status at the level of estimating treatment outcome in terms of mucosal healing. CRP is routinely used with suspected intestinal inflammation, but can reflect neutrophil challenge at many other organs (e.g., hepatic inflammation secondary to liver steatosis or metabolic syndromes). Elevated I-FABP, concomitant with elevated clinical inflammatory markers (e.g., TNF*α*, CRP), should in principle more accurately reflect inflammation (i.e., enterocytes damage or turnover) that can be attributed to the gut. Future studies are warranted to evaluate positive and negative predictive values using measures such as sensitivity and selectivity when I-FABP values are included. In the context of individual patient monitoring, the variability in the parameters in Table 1 is telling. For example, CD subject number 1 had the lowest CRP (0.6 mg/L was average for healthy subjects), but had the second highest HBI, while TNF*α* and I-FABP were intermediate for the group; I-FABP was above 75% percentile but below 95% percentile. CD subject number 4 had the highest I-FABP (well above 95% percentile) and HBI, but the lowest TNF*α*. No single parameter listed in Table 1 on its own would be able to be predictive of colonoscopy findings across all patients. The data suggests there could be added value in multiparametric assessments that include I-FABP. As a candidate clinical chemistry analyte, it is noteworthy that I-FABP was apparently not degraded during ~12 years of biobanking (−20°C).

The strongest I-FABP expression occurred in jejunum and ileum. According to a recent study by Thia et al. [17], CD disease activity is present in terminal ileum (L1, 45% of cases), ileocolon (L3, 19%), and upper GI (L4, 4%), implying that about 68% of CD cases can have ileum or upper GI involvement where I-FABP is expressed. Consistent with the claim stated herein that I-FABP could be used as a biomarker for upper/midgut mucosal healing in CD, Pelsers et al. [18] demonstrated that although I-FABP is expressed in a few different human organs, the highest expression occurred in the jejunum. Extending on the Pelsers et al. paper, it was found here that I-FABP immunoreactivity at ileum equals that of the jejunum, with significant expression in the colon where most CD lesions occur. It is then conceivable that I-FABP could also be used to monitor UC treatment outcomes, perhaps in conjunction with sugar permeability tests that can rapidly assess tight junctional permeability [12, 19].

During the course of this study, preliminary work by Sarikaya et al. [20] reported that I-FABP could be diagnostic for CD. Bodelier et al. [21] found that I-FABP was not predictive for endoscopic disease activity in IBD. The I-FABP changes within individuals that temporally paralleled infliximab therapy seen in this study cannot be explained by random chance and align well with Sarikaya et al.'s study. However, due to preferential expression in the jejunum and ileum, serum (or plasma) I-FABP would be expected to be most reflective of involvement in these GI regions. I-FABP concentrations in healthy subjects were in line with the previous studies using the same kit [9]. Plasma values in CD in Bodelier et al. study [21], which used an in-house assay, were several times lower than serum values reported here. The control experiments herein offer an explanation in that I-FABP was lower in plasma than serum from same subjects on same occasion. A final determination on usefulness of I-FABP in monitoring CD should await improved methodologies, such as optimized preservation of samples and type of sample (e.g., plasma versus serum). At this time, we recommend that serum be used with protease inhibitor cocktail and that samples be frozen as early as possible after collection and thawed immediately before assay. Any remaining incongruities in endoscopy versus I-FABP (or permeability) tests could be explored for added value when combined.

## 5. Clinical Perspectives

- Circulating I-FABP parallels TNF*α* during infliximab treatment in active Crohn's disease (CD).
- I-FABP may be useful to monitor CD patients in terms of treatment outcome and/or mucosal healing/remission cycles.
- I-FABP fluctuations around the 75% percentile of healthy subjects may be a practical cutoff for monitoring CD.

## Figures and Tables

**Table 1:** Characteristics and baseline values of TNF*α*, I-FABP, CRP, and HBI for 10 CD patients included into the study. The average age was 40 ± 4 years of age, and 70% were male. Serum TNF*α* was 2.34 ± 0.22 ng/L, I-FABP was 2.07 ± 0.23 *μ*g/L, CRP was 11.7 ± 5.6 mg/L, and HBI score was 10.5 ± 2.2. Values are mean ± SEM.

Subject number	Age	Sex	Inflammation site	TNF*α* pretreatment (ng/L)	I-FABP pretreatment (*μ*g/L)	CRP pretreatment (mg/L)	HBI scores, pretreatment
1	34	M	Colon	2.18	1.75	0.6	20
2	48	F	Colon	1.90	1.62	6.0	11
3	58	M	Colon	2.32	2.32	20.0	2
4	34	M	Colon	1.41	3.70	4.0	22
5	45	F	Ileocecal	1.85	2.62	2.9	12
6	21	F	Ileocecal	2.73	2.01	60.0	10
7	20	M	Colon	2.38	1.10	1.2	14
8	50	M	Colon	1.76	2.27	6.3	5
9	36	M	Colon	3.82	1.46	7.6	8
10	58	M	Colon	3.03	1.80	8.1	1

**Table 2:** Immunohistochemistry scores for I-FABP and relative abundance by gut segment. Score is histological intensity. Photometric correction is a multiplier that adjusts visual scoring intensity values to stepwise changes in concentrations quantified photometrically by a spectrophotometer. Square meters (m^2^) of mucosal surface area were obtained from Helander and Fändriks [15]. Duodenum tissue was not available for this study, so the score was estimated from Besnard et al. [16]. Size correction (size corr.) was used to allow for distribution across the same segment (e.g., corpus is approx. 70% of the stomach). Relative abundance is apparent relative abundance of I-FABP in stated segment (score × area × size corr.), whereas corrected relative abundance replaces score with photometric correction. The last two columns give abundances as percent of the total GI abundance.

Segment	Score	Photometric correction	Area m^2^	Size corr.	Relative abundance	Corr. rel. abundance	% abundance	Corr. % abundance
Cardia	3	16	0.05	0.1	0.015	0.08	0.011	0.004
Fundus	2	4	0.05	0.2	0.02	0.04	0.015	0.002
Corpus	2	4	0.05	0.7	0.07	0.14	0.053	0.007
Duodenum	2	4	3.3	1.0	6.6	13.2	4.973	0.672
Jejunum	4	64	15.0	1.0	60.0	960.0	45.213	48.844
Illeum	4	64	15.0	1.0	60.0	960.0	45.213	48.844
Colon	3	16	2.0	1.0	6.0	32.0	4.521	1.628
Sum	N/A	N/A	35.45	N/A	132.705	1965.46	100	100

## Abbreviations

CD: Crohn's disease
CRP: C-reactive protein
F*x*: Follow-up serum sample one week after infliximab infusion, where *x* indicates follow-up visit number
GI: Gastrointestinal
HBI: Harvey-Bradshaw Index
IBD: Inflammatory bowel diseases
Inf*x*: Infliximab infusion serum sample, where *x* indicates treatment visit number
I-FABP: Intestinal fatty acid binding protein
TNF*α*: Tumor necrosis factor alpha
UC: Ulcerative colitis.
